# Iatrogenic Dental Trauma During Airway Management: A Case Report and Review of Preventive Strategies

**DOI:** 10.7759/cureus.110325

**Published:** 2026-06-05

**Authors:** Arunkumar Muthalu, Arthi Asokan, Mirthan Krish, Kavya Ganesh

**Affiliations:** 1 Department of Critical Care Medicine, Mahatma Gandhi Medical College and Research Institute, Sri Balaji Vidyapeeth (Deemed to be University), Puducherry, IND; 2 Department of Anaesthesiology, Sri Venkateshwaraa Medical College Hospital and Research Centre, Puducherry, IND

**Keywords:** airway management, airway obstruction, anesthesia complications, foreign bodies, intratracheal, intubation, laryngoscopy, magill forceps, tooth avulsion, tooth injuries

## Abstract

Dental trauma is a recognized complication of airway management during general anesthesia, particularly in patients with poor dentition. Dislodged teeth pose a significant risk of airway obstruction, aspiration, and subsequent pulmonary complications, with important medico-legal implications. We report the case of a 45-year-old male with multiple loose and missing teeth, scheduled for an uncemented bipolar hemiarthroplasty under general anesthesia following a polytrauma incident one week prior. Despite appropriate preoperative counseling and informed consent, a dental injury occurred during direct laryngoscopy. An upper central incisor fractured and was immediately retrieved. Subsequently, an upper lateral incisor became dislodged and could not be located on the initial laryngoscopic examination or the intraoperative chest X-ray. Systematic reassessment, including thorough suctioning and repeat airway inspection, revealed the tooth lodged in the pyriform fossa, which was successfully retrieved using Magill’s forceps. The patient remained hemodynamically stable throughout, with no evidence of aspiration or postoperative complications.

Dental injuries during anesthesia are multifactorial and more common in patients with pre-existing dental pathology. This report highlights the importance of a meticulous preoperative dental assessment, clear risk communication, and thorough documentation. It also underscores the need for a structured, persistent approach to locating and retrieving dislodged teeth, as they may not always be detectable on radiographic imaging. Iatrogenic dental trauma during airway management can be effectively managed with prompt recognition, systematic evaluation, and timely retrieval. Preventive strategies, careful airway handling, and adherence to a structured retrieval protocol are essential to minimize complications and medico-legal risk.

## Introduction

Dental injuries during the administration of General anesthesia represent an inadvertent complication, particularly during tracheal intubation in patients with pre-existing dental abnormalities such as damaged, loose, or malaligned teeth, which can complicate airway management and lead to dental injury, including tooth dislodgment [1-2]. These incidents may cause airway obstruction, aspiration of teeth leading to lung collapse or pneumonia, and, in severe cases, esophageal perforation or mediastinitis, potentially resulting in medico-legal consequences for the anesthetist [3-4]. Careful prevention and prompt management, including preoperative dental assessment, the use of protective guards or video laryngoscopes, and prompt replantation of avulsed teeth within 60 minutes, followed by consultation with a dental specialist, can effectively mitigate risks and prevent further complications [5-6].

We report a case of laryngoscopy-induced dental trauma in which a dislodged upper lateral incisor was noticed after intubation but was subsequently lost during retrieval attempts, evading detection on initial digital examination under laryngoscopy and on chest X-ray. The tooth was eventually located in the pyriform sinus after repeat examination and thorough nasopharyngeal suctioning and was successfully retrieved using Magill's forceps. Dental trauma commonly occurs during difficult laryngoscopy. However, we report this case because of the difficulty encountered in locating the missing tooth despite initial examination and radiographic evaluation. Ultimately, simple nasopharyngeal suctioning and a repeat airway examination facilitated successful retrieval. We would like to emphasize the importance of a systematic approach to preventing dental injuries, including thorough preoperative dental assessment, the use of protective strategies, and the use of video laryngoscopy when indicated. We also highlight the importance of prompt retrieval and replantation of the tooth if such injuries occur.

## Case presentation

A 45-year-old male was admitted after sustaining injuries in a road traffic accident and was diagnosed with a right neck of femur fracture along with L1 and L4 burst fractures of the spine. He had a history of hypertension for the past three years and was on regular treatment with the tablet amlodipine 5 mg once daily. A routine pre-anesthetic evaluation was performed. Airway examination revealed restricted neck extension of less than 25 degrees, a mouth opening of more than three finger breadths, and Modified Mallampati Grade III. All other laboratory investigations were within normal limits. Oral examination revealed poor dentition, with multiple carious teeth and loose central incisors exhibiting extensive dental decay.

Based on the preoperative assessment, the patient was classified as American Society of Anesthesiologists (ASA) Physical Status II. The lumbar fractures were undisplaced and were not associated with any sensory or motor deficits, and there was no clinical deterioration following the injury. Therefore, the orthopedic team planned conservative management for the spinal injuries. However, the patient was scheduled for an uncemented bipolar hemiarthroplasty under general anesthesia because of the concomitant lumbar spine fractures, which precluded neuraxial anesthesia.

As the patient had multiple loose teeth, including the central and lateral incisors, the risks of difficult laryngoscopy and potential tooth dislodgement were explained to both the patient and his attendants. After obtaining written informed consent in the patient’s native language, detailing the risks and benefits of anesthesia, the patient was shifted to the operating room. Standard ASA monitors were attached, preoxygenation was performed, and induction was achieved with 100 mg IV of propofol, followed by muscle relaxation with 100 mg IV of succinylcholine. Laryngoscopy revealed a Cormack-Lehane Grade III view. During intubation, the upper central incisor fractured, which was directly visualized and immediately retrieved under laryngoscopy, allowing successful placement of an 8 mm ID oral endotracheal tube on the second attempt with bougie assistance.

While withdrawing the laryngoscope, the upper lateral incisor became dislodged into the oral cavity but could not be located on initial direct laryngoscopic examination. A C-arm X-ray of the tracheal region and lung fields was performed, which revealed no visible foreign body in the parapharyngeal area or lungs. Subsequent examination with thorough nasopharyngeal suctioning identified the tooth lodged in the pyriform fossa, which was successfully retrieved using long Magill forceps. Post-procedure, the patient remained hemodynamically stable with adequate respiratory efforts. He was reversed, successfully extubated, and continued to maintain stable vitals, with no emesis or signs of aspiration. The retrieved tooth was handed over to the attendants, and the patient was routinely monitored until discharge, with no perioperative or postoperative complications reported.

## Discussion

Dental injury during general anesthesia is a well-recognized complication and represents one of the most common causes of anesthesia-related medico-legal claims [7]. The incidence varies widely but is significantly higher in patients with poor dentition, loose teeth, or dental prostheses [8-9]. Multiple factors contribute to dental trauma, including patient-related factors such as fragile dentition and anatomical variations, procedural challenges such as difficult laryngoscopy and repeated intubation attempts, and operator experience [9-10]. The design and type of laryngoscope blade also influence the risk, with excessive pressure exerted on the maxillary incisors representing a major mechanism of injury [11]. Loose or malaligned teeth pose a particularly high risk during airway manipulation. This is especially relevant in patients with periodontal disease and children with mixed dentition [12]. A structured preoperative dental evaluation is therefore essential and should be thoroughly documented, ideally using a dental chart [13].

Preventive strategies include stabilization or elective removal of loose teeth when feasible, or temporary securing using silk sutures in emergencies. The use of protective adjuncts such as bite blocks, dental guards, and video laryngoscopes can further reduce the risk of trauma [14]. Preoperative counseling and informed consent regarding the risk of dental injury are crucial, both for ethical practice and medico-legal protection. Post-intubation and post-extubation reassessment of dentition should also be routinely performed and documented. In cases of dental avulsion, immediate priorities include localization and retrieval of the missing tooth to prevent aspiration or ingestion [15]. Dental fragments may not always be radio-opaque, making direct visualization and systematic airway examination essential. Imaging modalities such as neck and chest radiographs, CT, and endoscopic techniques may be required when the tooth is not immediately identified [16].

In our case, despite initial failure to locate the dislodged tooth using laryngoscopy and radiography, persistent, systematic reassessment led to its identification in the pyriform fossa and successful retrieval using Magill’s forceps. In the worst-case scenario, failure to retrieve the second tooth could result in its aspiration into the airway, making removal more difficult and potentially hazardous. We emphasize thorough preoperative dental examination and preventive measures, such as securing loose teeth through elective dental intervention or, in emergencies, temporary stabilization using silk sutures around the gingival margins. Patients should always be informed of the risk of dental injury before intubation, dentures should be checked, and findings must be recorded after both intubation and extubation. In cases of avulsion, the anesthesiologist must promptly localize and retrieve any tooth or fragments and ensure they are handed over to the patient or attendants, thereby preventing medico-legal complications related to improper disposal.

A meticulous recovery protocol is crucial, including direct oral laryngoscopy, as dental fragments may not be radio-opaque, immediate neck and chest X-rays, high-resolution CT (HRCT) of the chest, soft tissue neck X-ray, or flexible esophagoscopy if esophageal lodgment is suspected. In this case, a dislodged upper lateral incisor was noticed post-intubation but was lost during attempted removal, evading detection on initial digital laryngoscopic examination and C-arm imaging of the chest and neck. The tooth was eventually located in the pyriform fossa after a repeat examination and thorough nasopharyngeal suctioning, and it was successfully retrieved using Magill's forceps.

This highlights the importance of vigilance, stepwise evaluation, and adherence to airway safety protocols in preventing serious complications such as aspiration or distal airway obstruction. We propose the following systematic stepwise measures for the prevention and management of dental injuries: (1) Assess the risk of dental injury during the preoperative phase and implement appropriate preventive strategies. (2) In the event of dental trauma resulting in complete avulsion, prompt reimplantation of the tooth should be considered when feasible. (3) Identify the location of any fractured tooth fragments and ensure their retrieval and removal. (4) Be aware that dislodged dental fragments may migrate not only into the airway and gastrointestinal tract but also into the nasopharynx. (5) Arrange for postoperative evaluation and follow-up by a dental specialist.

## Conclusions

Dental trauma during airway management remains a preventable yet clinically significant complication with important patient safety and medico-legal implications. This report underscores the critical importance of a comprehensive pre-anesthetic dental evaluation, meticulous documentation, and detailed patient counseling, particularly in individuals with poor dentition or loose teeth. Adopting preventive strategies, such as gentle laryngoscopy, minimizing pressure on the maxillary incisors, and considering alternative airway devices in high-risk patients, can substantially reduce the incidence of such injuries. Early retrieval is essential to prevent potentially serious complications, including aspiration, airway obstruction, and distal migration of dental fragments. Incorporating structured protocols or algorithms for prevention and retrieval into routine anesthetic practice can further enhance patient safety and reduce adverse outcomes during airway management.
